# The Characterization of Pastures by Grazing Cycle and Evaluation of Supplementation with Agro-Industry Co-Products on the Performance of Buffaloes in the Humid Tropics

**DOI:** 10.3390/ani14060879

**Published:** 2024-03-13

**Authors:** Kelly Cavalcanti Conor de Oliveira, Cristian Faturi, Alexandre Rossetto Garcia, Maria Regina Sarkis Peixoto Joele, Benjamin de Souza Nahúm, Welligton Conceição da Silva, Thomaz Cyro Guimarães de Carvalho Rodrigues, Éder Bruno Rebelo da Silva, José de Brito Lourenço-Júnior

**Affiliations:** 1 Postgraduate Program in Animal Science (PPGCAN), Institute of Veterinary Medicine, Federal University of Para (UFPA), Federal Rural University of the Amazônia (UFRA), Castanhal 68746-360, Brazil; kellyconor@hotmail.com (K.C.C.d.O.); thomazguimaraes@yahoo.com.br (T.C.G.d.C.R.); eder.b.rebelo@gmail.com (É.B.R.d.S.); joselourencojr@yahoo.com.br (J.d.B.L.-J.); 2 Institute of Animal Health and Production, Federal Rural University of the Amazônia (UFRA), Belem 66077-830, Brazil; cristian.faturi@ufra.edu.br; 3 Embrapa Southeast Livestock, São Carlos 13560-970, Brazil; alexandre.garcia@embrapa.br; 4 Embrapa Eastern Amazon, Belém 66095-903, Brazil; reginajoele@hotmail.br (M.R.S.P.J.); nahum@embrapa.br (B.d.S.N.)

**Keywords:** water buffalo, coconut cake, co-products, nutrition, palm kernel cake, protein fractions, agroforestry systems, pasture supplementation, performance of water buffaloes

## Abstract

**Simple Summary:**

The objective was to characterize the pastures by grazing cycle, as well as to evaluate the performance of buffaloes in intensive rotational grazing in a silvopastoral system in the eastern Amazon supplemented with agro-industry co-products in order to characterize the grazing cycles, the composition of the fractions and the carcass yield. Fifteen non-castrated, crossbred water buffaloes (Murrah × Mediterranean) were used. Forage protein varied: leaves contained 11.4% protein in the leaves and 50% FDA in the stems. The grass had higher amounts of B3/B2 proteins, which are less indigestible in leaves (17.16%). Forage production varied; the second cycle was better (leaf/stem 2.11). Despite a varied supplement intake, daily weight gain (1 kg/day) and carcass yield (49.23%) showed no differences, ensuring cost-effective, sustainable production. Incorporating supplements derived from coconut and palm kernel co-products enhances performance and carcass yield, comparable to standard supplements. This practice lowers production expenses, optimizes forage utilization, and enhances production chain sustainability, making it a recommended approach.

**Abstract:**

The objective was to characterize the pastures by grazing cycle, as well as to evaluate the performance of buffaloes in intensive rotational grazing in a silvopastoral system in the eastern Amazon supplemented with agro-industry co-products in order to characterize the grazing cycles, the composition of the fractions, and the carcass yield. Fifteen non-castrated, crossbred water buffaloes (Murrah × Mediterranean) were used. All animals used in the study were clinically healthy and weighed approximately 458 kg. The animals were grazed in a single group, and supplementation (1% of live weight—LW/day) was divided into three treatments: control (control—conventional ingredients); *Cocos nucifera* coconut cake (*Cocos nucifera*) (coconut cake—70%); and palm kernel cake (*Guinean elaeis*) (palm kernel cake—70% palm kernel cake). The chemical composition of the forage is different in each part of the plant, with higher protein values in the leaves (11.40%) and higher acid detergent fiber (ADF) values in the stems (50.03%). Among the ingredients of the supplement, corn has the highest percentage of indigestible protein (35.57%), most of the protein in palm kernel cake is B3 (49.11%), and in Coco, B2 (51.52%). Mombasa grass has a higher percentage of B3 and B2 proteins; the indigestible fraction is lower in the leaves (17.16%). The leaf/stem ratio also varied between grazing cycles, being better in the second cycle (2.11%) and with an overall average of 1.71. Supplement consumption varied between cycles and was higher in the control treatment, with an overall mean of 4.74. There was no difference in daily weight gain and carcass yield, with an average of 1 kg/day and 49.23%, respectively. Therefore, it can be concluded that including supplements based on by-products from the coconut and palm oil agro-industries promotes performance and carcass yields compatible with conventional supplements. Their use reduces production costs, optimizes the utilization of forage mass, enhances the sustainability of the production chain, and, therefore, is recommended.

## 1. Introduction

In Brazil, especially in the Amazon region, water buffalo farming is significantly increasing. This widely adopted practice is due to the favorable climatic conditions of the environment. Furthermore, water buffaloes are highly valued for adapting to lower-quality forage without compromising their productive performance, making them a suitable choice for the region [1]. The advantages of using water buffaloes include their ability to digest fibers that are difficult for cattle to digest, as well as their better ability to retain water and different nutrients such as proteins and minerals, making them more adaptable animals [2].

The supplementation of ruminants in an extensive system aims, in addition to increasing efficiency in the use of pastures, to correct nutritional deficiencies in the pasture, to increase the intake of digestible dry matter, and to maintain animal performance [3]. This strategy is mainly used in places where the supply and quality of pasture fluctuates throughout the year, such as the eastern Amazon. In this region, there is a period with a high rainfall index and food availability and a dry period during which the quality and quantity of forage are reduced [4].

However, supplementation raises production costs, especially using conventional ingredients such as corn and soy [5,6]. In this sense, the search for unconventional feedstuffs with a low commercial value, such as agro-industry co-products, can make the strategy viable [7]. Its use as an ingredient in diets allows for adjustments in the feed supply throughout the year, increases the support capacity of pastures, gives an adequate destination to this biomass, and decreases the need for deforestation to obtain new livestock areas [8,9,10].

The northern region of Brazil, where the eastern Amazon is located, is the largest producer of palm oil from dendê and the second largest producer of coconuts [11]. Cakes are also obtained as a co-product of these two crops after mechanical oil extraction. This biomass, as a function of protein (up to 22.8% of crude protein), energy (up to 16.5% of ether extract), and fibers (up to 61% of fiber in neutral detergent) has been studied as an ingredient in the formulation of ruminants’ diets [12,13,14,15,16]. For this biomass, it is expected to find protein digestibility values in the range of 50% to 80% and energy digestibility values in the range of 60% to 80% in some studies.

In various regions, climatic conditions, different species, breeding methods, and management systems have already been investigated concerning co-products [17,18]. Coconut cake consumption improved milk production and increased cattle profitability; goats that consumed 10% coconut cake in their diet had a higher average daily weight gain and the cost of feed per weight gained was low; and palm cake increased consumption and zootechnical performance without compromising nutrient digestibility and feed efficiency in goats.

The diet supplementation of water buffalo cows with palm kernel cake did not influence fiber degradability and was recommended up to 1% of body weight [10,15,18]. The average composition of palm kernel cake produced in the Metropolitan Mesoregion of Belém and the northeastern region of Pará, where variations occurring among processing units are 92.96%, 11.96%, 27.17%, 12.09%, and 72.28%, respectively, for dry matter, crude protein, crude fiber, ether extract, and total digestible nutrients [10].

The agroforestry system was used to provide shade for the animals to provide thermoregulation to the animals. The study of water buffalo nutrition is essential due to the unique productive characteristics of this species. Water buffaloes play a crucial role in milk and meat production. Their characteristics include a remarkable ability to adapt to challenging environments, such as wet and marshy areas, where traditional cattle may not thrive. Additionally, water buffaloes are known for their efficiency in utilizing low-quality forage, making them a valuable food source in regions with limited resources. For these reasons, the choice was made to work with water buffaloes in this article [19,20]. Thus, we hypothesize that the inclusion of supplements based on co-products (coconut and palm kernel cakes) can offer a performance comparable to the conventional ones. The objective was to characterize the pastures by grazing cycle, as well as to evaluate the performance of buffaloes in intensive rotational grazing in a silvopastoral system in the eastern Amazon supplemented with agro-industry co-products in order to characterize the grazing cycles, the composition of the fractions, and the carcass yield.

## 2. Material and Methods

### 2.1. Ethical Aspects

The experimental procedures were previously approved under protocol number 007/2015 by the Ethics in Animal Use Committee of Embrapa Amazônia Oriental, which is a company linked to the Brazilian Ministry of Agriculture, Livestock and Supply.

### 2.2. Experimental Area

**Figure 1:**
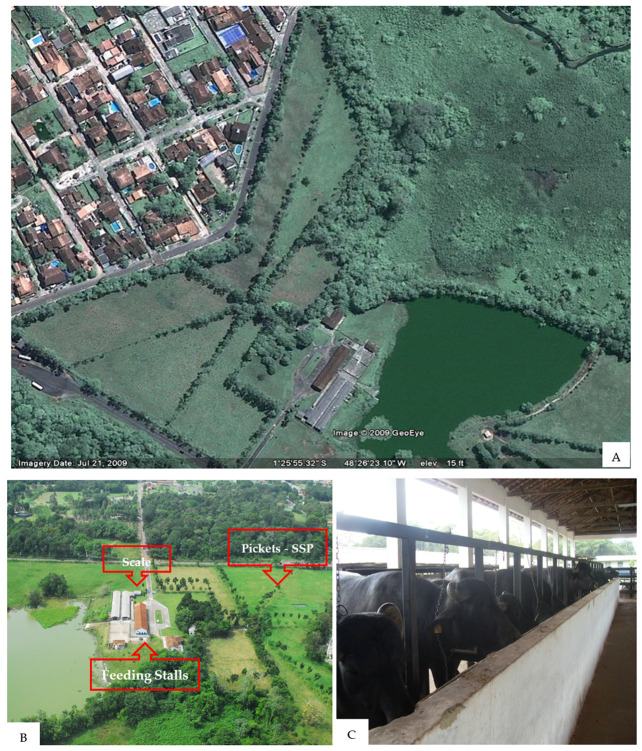
Experimental area. (**A**) Experiment area. (**B**) Description of the main areas of the experiment. (**C**) Animals separated into individual feeding pens. SSP = silvopastoral system.

The research was carried out at the Animal Research Unit “Senador Álvaro Adolpho”, belonging to Embrapa Amazônia Oriental, in Belém, Pará (1°28″ S and 48°27″ W) (Figure 1A–C). The climate type is Afi (Köppen classification), with an average rainfall of 3001.3 mm/year, with the rainiest period being from December to May and the least rainy period from June to November. The average annual temperature is 26.4° C, with an average relative humidity of around 84% and an annual insolation of 2338.3 h/year [21]. The soil is of the Yellow Latosol type and stony phase, an analysis of which revealed the following composition: pH = 4.5, P = 1 ppm, K = 14 ppm, Ca + Mg = 1.6 meg/100 g, and Al = 2 meg/100 g.

The research took place in a silvopastoral system, implemented in an area of 5.4 ha, with five pickets of electrified fences, and interconnected by a central common area equipped with an automatic drinking fountain and trough for mineral supplementation. In the area, African mahogany (*Khaya ivorensis*) and Indian neem (*Azadirachta indica*) trees have been planted (over the last ten years) 4 m apart, shading about 20% of the area. The system has Mombasa grass (*Panicum maximum* cv Mombasa) managed in an intensive 30-day grazing cycle rotation, with 6 days of occupancy and 24 days of rest. The final and initial stocking rates were, respectively, 3.0 and 4.5 AU. The entire area was prepared for pasture implantation using tractors (plowing, harrowing, clod-breaking, and leveling) and fertilization with 300 kg/ha of Arad (reactive natural phosphate), with 33% P2O5.

### 2.3. Animals and Diets

The experiment was carried out between the months of April and October of 2009, with the first month for the adaptation of the animals to the diets, management, and facilities and five months for data collection. Fifteen non-castrated, crossbred (Murrah and Mediterranean), male water buffaloes in the finishing phase with an average weight of 458 kg were used. The animals had a body score of 3, which is considered adequate on a scale of 1 to 5. The water buffaloes were divided into three groups, each containing five experimental animals. The animals were weighed after a 14 h water and feed fast at the beginning of the research and at the end of each grazing cycle, and the feed supplementation was adjusted after each weighing. Sanitary prerogatives concerning vaccinations and parasite control followed Lau’s indications.

In addition to grazing, the animals had access to water and mineral salt ad libitum, as well as to supplementation prepared with agro-industrial co-products, the chemical compositions of which are shown in Table 1.

Supplementation was provided every morning in individual pens when the animals were led to the covered shed. The total offered and the leftovers were weighed to estimate each individual consumption. The supplements were formulated to be isoproteic, with 19% crude protein (CP) in the three treatments (diets): control (control—based on corn and soy)—pasture + supplement; coconut cake (Coco—70% coconut cake)—pasture + supplement; and palm kernel cake (palm kernel cake—70% palm kernel cake)—pasture + supplement (Table 2).

Thus, the total diet consisted of roughage (ad libitum in intensive rotational grazing), supplement (1% of live weight), water, and mineral salt ad libitum. The rations were formulated according to the Nutrient Requirements of Dairy Cattle—NRC [22], and the chemical composition is in Table 1.

### 2.4. Forage Production and Nutritional Value

#### 2.4.1. Forage Production

The available forage mass was estimated by cutting the height at 5 cm from the ground, using a frame made of wood measuring 0.25 m^2^ (0.50 m × 0.50 m). The collection was carried out at eight points of the paddock, chosen at random, at the entrance and exit of each experimental paddock throughout the research. The collected samples were stored in identified bags and taken to the laboratory for processing and analysis. The material was separated into green matter (stems and leaves) and dead material. Afterward, the different fractions were dried in an oven with air circulation at 55 °C for 72 h and weighed again, according to Silva and Queiroz [23].

#### 2.4.2. Nutritional Value of Forage and Diet

After drying, the samples were crushed in a Wiley-type mill (1 mm sieve) for the determination of dry matter (DM), neutral detergent fiber (NDF), acid detergent fiber (ADF), lignin [23], and crude protein (CP) (Kjeldahl—[23]). In the protein fraction, the nitrogen fractions were also determined: “A”, consisting of non-protein nitrogen compounds; “B1”, for soluble proteins, rapidly degraded in the rumen; “B2”, insoluble proteins, with an intermediate degradation rate; “B3”, insoluble proteins, with a slow degradation rate in the rumen; and fraction “C”, consisting of insoluble proteins, indigestible in the rumen and intestines [24]. The constituents of the fibrous fraction were determined according to Silva and Queiroz [23].

#### 2.4.3. Performance and Carcass Yield

At the end of the experiment, the animals were weighed while fasting and taken to the slaughterhouse. Before slaughter, they were weighed again to obtain the carcass yield. The carcass yield was obtained after slaughter, respecting the handling and humane slaughter procedures. Performance was assessed by weighing the animals individually on the first experimental day, after the adaptation period, and on the last day. They were always performed in the morning, after the solid fasting period (14 h). Thus, total weight gain (TWG) was determined by the difference in final and initial body weight, and the average daily gain (ADG) was determined by the TWG divided by the number of days of the research.

### 2.5. Statistical Analysis

**Figure 2:**
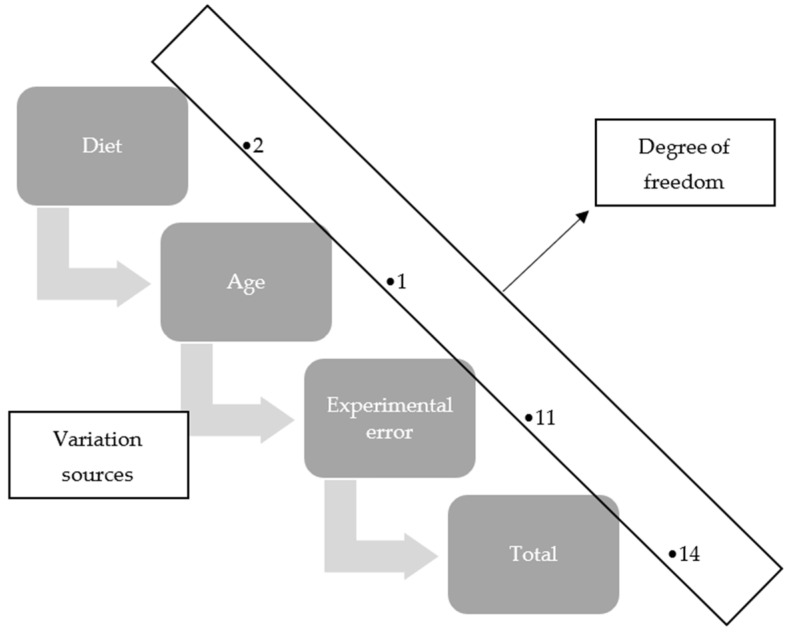
The response variables were analyzed in an experimental design in randomized blocks, with two blocks, three treatments, and five replications.

The response variables were analyzed in an experimental design in randomized blocks, with two blocks (ages), three treatments (type of supplement), and five replications (animals) (Figure 2). The data obtained from the animals, such as intake and weight gain, were subjected to an analysis of variance using Proc GLM, with a model including the fixed effects of the supplement type, grazing cycle, block, and interaction between supplement type and grazing cycle, and the random effect of the residue; whereas, for the final weight and carcass yield data, the model only includes the fixed effects of the type of supplement and block and the random effect of residue. The comparison of means using the “*t*” test with the aid of the SAS statistical package [25,26] at 5% probability is visualized as follows.

## 3. Results

### 3.1. Characterization of Plant Parts and Grazing Cycle

The percentage of dry matter increased with each grazing cycle (Table 3): in the evaluation of the whole plant (WP), it obtained 25.04% in the first cycle and 32.08% in the fifth cycle; in the leaves, it ranged from 23.83% to 36.04%; and in the stem, the variation was from 19.46% to 33.73%.

Unlike DM, the protein percentage did not have a gradual variation: in the WP, the lowest value was verified in cycle 3 (5.93%) and the highest in cycle 5 (8.69%); in the leaves, the variation was low, oscillating between 11.02% (cycle 2) and 11.85 (cycle 1); and in the stem, it ranged from 4.24% (cycle 2) to 5.51 (cycle 5). The highest average (11.40%) was also observed in the leaves.

The NDF variation also did not show a gradual behavior: in the WP, it ranged from 64.59 (cycle 5) to 71.81% (cycle 1); in the leaves, the variation was between 65.02% (cycle 3) and 69.83% (cycle 2); and in the stem, with a more significant variation, it ranged from 61.19% (cycle 1) to 90.43% (cycle 5). The NDF averages in the WP (68.97%) and leaves (68.04%) were close, but the highest average was in the stem (74.74%).

The ADF ranged from 43.48% (cycle 5) to 51.63% (cycle 3) in the WP evaluation, from 36.65% (cycle 3) to 42.91% (cycle 2) in the leaves, and from 47.06% (cycle 4) to 50.03% (cycle 2) in the stem. The average percentage of ADF (WP) was 47.83 but was 39.25% in the pasture leaves.

The lignin averaged 9.0 (WP), 9.2 (leaves), and 8.52 (stem). The WP analysis showed variation between 7.03 (cycle 2) and 11.84 (cycle 3); in the leaves, it was between 6.42 (cycle 1) and 14.29 (cycle 5); and in the stem, from 5.92 (cycle 4) to 11.01 (cycle 3).

### 3.2. Protein Fraction Composition

By fractionating the protein, we found that, except for palm kernel cake, which has a higher percentage of B3 (49.11), the ingredients have higher values of the B2 fraction: corn (61.78), soybeans (67.52), wheat (49.08), and Coco (51.52) (Table 4). The values referring to fraction C (insoluble and indigestible protein) were higher in corn (35.57), followed by soy (21.35), palm kernel cake (18.87), Coco (11.39), and wheat, with the lowest percentage (4.80). In the B1 fraction (soluble protein of rapid degradation in the rumen), the highest value was observed in wheat (15.29), followed by palm kernel cake (13.14), soybean (9.12), Coco (2.06), and corn, with the lowest percentage of this protein (0.04).

On average, the protein fraction most found in the forage is B3, including in the WP (39.87), the leaf (52.27), and the stem (35.08) (Table 4). The second most abundant fraction in the WP (28.61) and leaves (24.22) is B2, but in the stem, it is the A fraction (29.45). The soluble and indigestible protein (C) is highest in the stem (28.43), followed by the WP (26.04) and leaves (17.16). The protein fraction that rapidly degrades in the rumen varies greatly between grazing cycles but is found in the stem at the highest percentage (3.44), followed by the leaves (3.07) and the WP (2.91).

### 3.3. Forage Mass per Hectare and Animal Live Weight

The average forage mass per hectare across the grazing cycles was approximately 6.47 kg. The highest production (7.247 kg) was recorded during the third cycle, while the lowest volume (4.926 kg) occurred in the final period (Table 5).

Thus, with animals grazing, there was a reduction of 3.27 kg of forage corresponding to animal weight changes between cycle 1 and cycle 5 (Table 6).

### 3.4. Leaf/Stem Ratio between Grazing Cycles

The percentage (%) of leaves averaged 38.43, with a maximum in the fifth cycle (42.96) and a minimum in the first cycle (34.81) (Table 6). The stem averaged 23.02, with a maximum in the first (25.70) and a minimum in the second (17.96) cycle. As for dead matter, it obtained an average of 38.54, with a maximum in the second (43.54) and a minimum in the last cycle (33.06). The leaf/stem ratio was lower in the first cycle (1.41), higher in the second cycle (2.11), and the average of the five cycles was 1.71.

### 3.5. Consumption, Weight Gain, and Carcass Yield

There was no difference in supplement consumption during the first cycle, with an average of 3.35 kg/animal (Table 7). In the second grazing cycle, animals from the control treatment group had the highest consumption (3.93), followed by animals from the palm kernel cake (3.50) and Coco (3.35) treatment groups. Consumption, in the third cycle, was also higher in the control treatment group (5.24), followed by animals from the palm kernel cake (4.62) and Coco (3.13) treatment groups. In the fourth cycle, the same behavior occurred, with higher consumption in the control treatment group (5.34), palm kernel cake treatment group (4.97), and then the Coco treatment group with the lowest consumption (2.59). In the last cycle, again, the lowest consumption was observed in the Coco treatment group (2.71), intermediate in the palm kernel cake group (4.65), and highest in the control group (5.78). Furthermore, the highest value was found in the fifth cycle for the control group (5.78 kg), while the lowest was recorded in the Coco group in the same cycle (2.71 kg).

Among the treatments and when evaluating grazing cycles, the control group exhibited an average consumption of 4.74, with the highest and lowest consumptions occurring in the fifth and first cycles, respectively (Table 7). In the Coco treatment, the average consumption was 2.99, with the highest and lowest consumptions observed in the second and fourth cycles, respectively. The palm kernel cake treatment had an average consumption of 4.24, with the highest intake observed in the fourth cycle (4.97) and the lowest in the first (3.43). Regardless of the supplementation provided or the grazing cycle, there was no significant difference in the animals’ weight gain (Table 8). The average weight gains were 0.94, 1.08, 1.01, 1.06, and 0.99 in the first, second, third, fourth, and fifth cycles, respectively. The mean weight gains per treatment were 1.01 for the control group, 0.97 for the Coco group, and 1.03 for the palm kernel cake group.

Supplements also did not influence the final weight or carcass yield (Table 9). The average final weight was 614.4 kg and the average carcass yield was 49.23 (%).

## 4. Discussion

### 4.1. Pasture Characterization by Grazing Cycle

Characterizing the pasture is fundamental for its maintenance and the performance of the animals [27]. With these data, adjustments can be made in pasture management, changes in the number of rotation days, and the composition of the animal supplements. The variation in the composition is normal because from one cycle to the next, there are not the same conditions, for example, luminosity, temperature, or rainfall [3]. In addition, there is movement (trampling) and feeding of the animals, which can be more or less intense along the paddock. The frequency and intensity of defoliation influence the production of dry and green matter and the chemical composition of *Panicum maximum* cv. Mombasa and proper management can provide greater forage productivity and quality, regrowth vigor, greater forage utilization efficiency, remarkable tissue renewal, and a more favorable canopy structure for grazing [28].

DM, ADF, and lignin values were above those reported in other studies, which varied, respectively, from 13 to 25, 38 to 44, and 7 to 8.6 [29]. Moreover, the values of CP, NDF, and lignin were below the findings in other studies, where the variation was from 8 to 10% (CP) and 70 to 74 (NDF) [30]. This reinforces the importance of characterizing the material and the variations resulting from the particularities of each region. The observed variations in NDF content among different plant parts, including the WP, leaves, and stems, across various cycles, indicate dynamic changes in fiber composition throughout the plant’s growth cycle. The WP’s NDF content fluctuated, with the highest level in cycle 1 and the lowest in cycle 5, resulting in an average NDF of 68.97. Leaves exhibited similar variations but maintained a slightly lower average NDF of 68.04 compared to the WP. In contrast, stems displayed the most significant variation, with cycle 5 featuring the highest NDF content and an overall average NDF of 74.74. These differences reflect the varying fiber composition in plant tissues during different growth stages and under changing environmental conditions, with stems typically being the most fiber-rich part due to higher cellulose, hemicellulose, and lignin content. These findings underscore the importance of considering NDF variations when evaluating plant materials’ suitability and nutritional value for diverse applications, such as animal feed or biomass production.

Higher levels of dry matter, acid detergent fiber, and lignin in pastures play a crucial role in the diet and performance of animals, particularly for ruminants. A greater concentration of dry matter results in more nutritionally dense pastures, helping to meet livestock’s energy and protein requirements. However, a proper balance with appropriate levels of acid detergent fiber is essential to ensure efficient digestion and promote gastrointestinal health. As for lignin, its presence in moderate amounts is necessary for plant structure, but excesses can make the nutrients contained in plant cells less accessible. Therefore, the importance lies in optimizing these elements to ensure that pastures provide high-quality feed, promoting the health and productivity of animals.

### 4.2. Protein Fraction Composition

For ruminants, crude protein can be divided into five fractions that show the non-protein fraction, different levels and times of rumen digestibility, or indigestibility associated with lignin and highly resistant to microbial and enzymatic degradation [24]. This methodology for assessing feed for ruminants has been used mainly in the evaluation of new ingredients, with the aim of characterization, efficiency in formulating diets, and the maximization of the use of nutrients by animals [31,32,33,34].

Coconut and palm kernel cake cakes have a lower percentage of protein fraction C, the one that is insoluble and indigestible in the rumen and intestines when compared to corn and soy (conventional ingredients); they also have low values of fraction A (non-protein nitrogenous compounds), a positive finding. Even in comparison with corn and soybeans, they also have higher levels of the B1 fraction (soluble proteins, quickly degraded in the rumen), which also reflects the quality of the material.

As the nutritional value of a feed does not depend exclusively on its nutrient content but also on the extent of ruminal degradation, it was observed that the sum of the digestible fractions (B1, B2, and B3) was greater in the coconut cakes (88%) and palm kernel cake (81.11) than in corn (64.45%) and soy (76.64), the conventional ingredients. The values found are compatible with other works, where the protein degraded in the rumen is 75–80% for soybeans and from 52 to 65% for corn [22,35,36,37]. However, there is also a comment on digestible protein’s amino acid composition and the investigation’s importance [37,38].

### 4.3. Forage Mass and Animal Live Weight

Several factors, such as seasonal attributes, pasture management, and grazing intensity, influence the forage production per hectare and the animal’s live weight [39]. Having control over the amount of available mass is extremely important. Therefore, the works use this mechanism, as this information is crucial for management adjustments (stocking rate and time) and the maintenance of animal performance, as well as providing enough time for the recovery of each paddock [40,41,42]. Possibly, the forage mass needed to be increased for the animal load in all cycles since it was reduced throughout the research. This may suggest that the animals’ gain was due to supplementation, with a possible substitutive effect. For Santos et al. [43], supplement energy levels are a tool for pasture management, reducing forage consumption and increasing stocking rate.

According to this hypothesis, some animals may have a higher consumption of forage due to limitations in the acceptance of supplements, which may also be associated with the palatability of the feed. For ruminants, a change in just one sensory characteristic of the food, such as taste, reflects a change in consumption [44,45]. Melo Lisboa et al. [46] reported a reduction in consumption without altering performance when palm kernel cake was used in cattle diets.

### 4.4. Leaf/Stem Ratio between Grazing Cycles

In addition to being the plant organ responsible for photosynthesis, the leaf is the primary source of nutrients for ruminants in grazing systems [47,48]. The higher the leaf/stem ratio, the more significant proportion of forage was left and, consequently, a higher protein content, digestibility, and consumption, and there was a better adaptation to management [49,50]. In addition to the individual characteristics of each forage species, luminosity, temperature, humidity, and water and nutrient availability can influence this relationship, making it essential to know the responses of each plant to the environment and management to adopt compatible practices [51].

Despite the variations between the grazing cycles, the average 1.71 for the leaf/stem ratio is favorable for the grass, as it deals with animals receiving supplementation. The data suggest that the pasture had a high percentage of leaves during the research and that the rest period may have been sufficient for its recovery under the management adopted. Gomide et al. [52] and Cândido et al. [53] evaluated the periods of neglect for this grass, and at 37 days, they observed a ratio of up to 0.92. The ratio varied between 1.7 and 4.3 and 0.92 and 2.93 in these cited works, respectively.

### 4.5. Consumption, Weight Gain and Carcass Yield

Food intake is the way in which animals acquire the necessary nutrients to maintain metabolic activities, growth, and performance [54]. Thus, measuring consumption, especially when including a new ingredient, becomes essential for supply adjustments and inclusion in diets. Despite the supply of concentrate having been adjusted proportionally to the weights, it was observed that the consumption of the control treatment was increasing, uniform in the palm kernel cake treatment group, and decreasing in the coconut treatment group. The behavior observed in animals on the Coco diet was reported by da Costa Braga et al. [55], evaluating levels of inclusion of this co-product in the diet when there was a reduction in consumption, with an increase in the ingredient’s inclusion. The increase in fat content consequent to greater inclusion may have influenced consumption, as the diet had a large proportion of concentrate (60%). Souza Junior et al. [56] also found a decreasing effect by way of verifying inclusion levels, whose lowest consumption was in the maximum inclusion of coconut cake (1.2% of BW).

The fact that animals fed with co-products did not follow the increase in consumption (observed in the control) may be related to the acceptability of the particular ingredient. Animals supplemented with palm oil maintained consumption levels from the third cycle, while in the second and fourth cycles, they were similar to the control treatment group. Ferreira et al. [57] and de Melo Lisboa et al. [46] observed a reduction; Rodrigues et al. [5] and Amaral-Júnior et al. [10] observed an increase in consumption; and Galvão et al. [58] and Salt et al. [59] found no difference. These variations in consumption may be related to the level of inclusion of fat and fiber content in the material used; as they are co-products, there is no homogeneity in the composition.

Assessing weight gain is a direct way to observe a diet’s efficiency. Despite the variations in supplement consumption and the diets with co-products obtaining lower intake than the control, the animals’ weight gain did not change. Thus, we suggest that the amount of nutrients ingested by the animals, regardless of the treatment, was equal and sufficient for weight maintenance and gain. The similarity in weight gain may be related to the compensation of the animals (Coco and Palm kernel cake) with forage consumption. Another hypothesis is that the energy density of supplements containing co-products was higher due to the increase in fat. The ether extract in palm kernel cake ranges from 5.7 to 13.55% and in coconut cake between 17.08 and 26.43 [5,10,57,60,61,62].

The weight gain obtained (±1.0 kg/day) is considered high when compared to the averages observed in the modal creation systems of the Amazon, which vary from 0.42 to 0.98 kg/day. The animals produced in this region, almost entirely in an extensive system, have low average weight gain and late slaughter [62]. Gains remained similar between treatments and grazing cycles, suggesting a possible efficiency of the management adopted, with the maintenance of gain during the period. This is another important fact, considering that one of the main problems of livestock in the Amazon is the oscillation in the quantity and quality of food, leading to the animals’ irregular performance. Carcass yield values were higher than the water buffalo averages in an extensive system in tropical regions, reinforcing the possibility of adopting the strategy [62,63].

It is worth mentioning that the environment influences the energy expenditure and performance of the animals. The microclimate promoted by the silvopastoral system, through shading (20% of the area), positively influenced the performance of the animals, requiring less energy expenditure thanks to thermoregulation. This condition has already been reported in studies in the Amazon, the silvopastoral system, and several ruminant species [64,65,66,67,68,69].

Recent findings could be examined in conjunction with physiological parameters such as core temperature, heart rate, respiratory rate, surface temperature, and weather conditions. This approach would enable a comprehensive understanding of the impacts of a specific production system on animal performance [70,71,72,73,74,75,76,77,78,79,80].

## 5. Conclusions

Significant variations in the chemical composition of the pasture were observed across different grazing cycles, closely related to the plant’s maturity and the composition of its various parts. Additionally, protein fraction analyses indicated an intermediate degradation rate in the animal’s rumen. Forage production remained consistent, with the third grazing cycle showing remarkable results. There were no significant differences in animal weight gain, irrespective of the type of supplementation provided or of the grazing cycle, and the carcass yield remained relatively stable. Consequently, the results suggest that supplementation with co-products such as coconut and palm kernel cakes promotes performance and carcass yield similar to conventional supplements. Thus, it emerges as a viable and recommended option.

## Figures and Tables

**Table 1:** Chemical compositions of supplements (%) and supplement ingredients.

	DM	CP	NDF	ADF	EE
Ingredients					
Shredded corn	87.62	8.04	13.98	8.30	3.84
Soybean meal	86.27	46.15	24.69	19.88	3.24
Wheat bran	86.42	15.45	40.50	12.12	3.46
Palm kernel cake	92.92	11.23	78.03	44.45	15.44
Coconut cake	93.54	21.40	54.73	37.04	11.08
Supplements					
Control	87.13	18.46	19.83	11.65	3.64
Coco	91.63	18.21	45.42	28.84	8.87
Palm kernel cake	90.97	16.95	63.87	35.84	11.82

DM = dry matter; CP = crude protein; NDF = neutral detergent fiber; ADF = acid detergent fiber; EE = ether extract.

**Table 2:** Proportion of components in each experimental diet.

Composition %		Treatments	
	Control	Coco	Palm Kernel Cake
Shredded corn	63	19	2
Coconut cake	-	70	-
Palm kernel cake	-	-	70
Soybean meal	25	0	15
Wheat bran	12	11	13

**Table 3:** Chemical composition (%) of pasture (*Panicum maximum* cv. Mombasa): whole plant (WP), leaf (L), and stem (S), by grazing cycle.

Plant	DM	CP	NDF	ADF	Lignin
WP 1	25.04	7.62	71.81	46.94	9.66
WP 2	29.48	6.36	69.01	49.12	7.03
WP 3	30.78	5.93	68.82	51.63	11.84
WP 4	31.01	6.78	70.64	47.97	8.66
WP 5	32.08	8.69	64.59	43.48	7.80
Averages	29.68	7.26	68.97	47.83	9.0
L 1	23.83	11.85	67.19	39.77	6.42
L 2	24.31	11.02	69.83	42.91	8.64
L 3	26.00	11.23	65.02	36.65	8.27
L 4	31.21	11.23	68.75	38.04	8.39
L 5	36.04	11.66	69.39	38.87	14.29
Averages	28.28	11.40	68.04	39.25	9.2
S 1	19.46	5.50	61.19	48.22	8.38
S 2	20.88	4.24	69.24	50.03	6.81
S 3	23.16	4.44	64.12	47.87	11.01
S 4	32.28	4.44	88.71	47.06	5.92
S 5	33.73	5.51	90.43	49.25	10.48
Averages	25.90	4.83	74.74	48.49	8.52

DM = dry matter; CP = crude protein; NDF = neutral detergent fiber; ADF = acid detergent fiber. WP = whole plant.

**Table 4:** Protein fractionation (%) of the supplement and pasture (*Panicum maximum* cv. Mombasa) in the whole plant (WP), leaf (L), and stem (S) by grazing cycle.

Ingredient	A	B1	B2	B3	C
Corn	0.00	0.04	61.78	2.63	35.57
Soja	9.50	9.12	67.52	0.00	21.35
Trigo	23.65	15.29	49.08	29.69	4.80
PKC	0.12	13.14	18.86	49.11	18.87
CC	5.38	2.06	51.52	34.42	11.39
Forage	%				
WP 1	23.99	3.58	34.35	41.41	16.66
WP 2	11.24	0.00	29.58	48.22	23.32
WP 3	3.83	7.35	22.25	37.01	32.15
WP 4	10.28	3.56	27.50	34.48	31.25
WP 5	20.69	0.06	29.38	38.21	26.81
Averages	14.01	2.91	28.61	39.87	26.04
L 1	21.81	6.50	28.18	47.93	14.28
L 2	18.37	0.00	22.64	43.24	28.84
L 3	29.40	0.00	29.18	58.63	11.31
L 4	23.26	0.00	21.02	62.72	13.19
L 5	22.42	8.83	20.07	48.84	18.18
Averages	23.05	3.07	24.22	52.27	17.16
S 1	36.78	0.04	42.08	26.30	23.09
S 2	17.79	5.83	17.58	35.38	34.99
S 3	31.28	6.21	25.04	31.20	28.60
S 4	31.23	0.06	24.94	37.51	28.56
S 5	30.15	5.04	14.93	45.00	26.92
Averages	29.45	3.44	24.91	35.08	28.43

PKC = palm kernel cake; CC = coconut cake; fractions: “A”, consisting of non-protein nitrogen compounds; “B1”, for soluble proteins, rapidly degraded in the rumen; “B2”, insoluble proteins, with intermediate degradation rate; “B3”, insoluble proteins, with a slow degradation rate in the rumen; and fraction “C”, consisting of insoluble proteins, indigestible in the rumen and intestines.

**Table 5:** Forage mass and dry matter production of Mombasa grass per animal throughout the grazing cycles.

Grazing Cycle	Forage Mass (kg/ha)	Dry Matter/Live Weight (kg DM.100 kg LW^−1^)
First	6.916.10	6.04
Second	6.994.50	4.74
Third	7.247.50	4.74
Fourth	6.268.50	3.77
Fifth	4.926.50	2.77
Average	6.470.61	4.41

DM = dry matter; LW = live weight.

**Table 6:** Percentage of forage parts in grazing cycles.

Grazing Cycle	Leaf	Stem	Dead Matter	L/S Ratio
First	34.81	25.70	39.49	1.41
Second	38.51	17.96	43.54	2.11
Third	35.05	23.33	41.62	1.50
Fourth	40.83	24.11	35.01	1.71
Fifth	42.96	23.98	33.06	1.83
Average	38.43	23.02	38.54	1.71

L/S Ratio = leaf/stem ratio.

**Table 7:** Average daily supplement intake (kg) per grazing cycle.

Grazing Cycle	Control	Coco	Palm Kernel Cake	Averages
First	3.43 Da	3.18 Aa	3.43 Ba	3.35
Second	3.93 Ca	3.35 Ab	3.50 Bab	3.59
Third	5.24 Ba	3.13 Ac	4.62 Ab	4.33
Fourth	5.34 Aa	2.59 Bb	4.97 Aba	4.30
Fifth	5.78 Aa	2.71 Bc	4.65 Ab	4.38
Averages	4.74	2.99	4.24	-

SEM = 0.2803; *p* = 0.0001 (interaction); means followed by capital letters (column) differ by the “*t*” test (*p* < 0.05). Means followed by lowercase letters (line) differ by the “*t*” test (*p* < 0.05).

**Table 8:** Average daily gain (kg) per grazing cycle and period.

Grazing Cycle	Treatments			Average
	Control	Coco	Palm Kernel Cake	
First	0.86	1.04	0.94	0.94
Second	1.03	1.16	1.04	1.08
Third	0.93	1.00	1.09	1.01
Fourth	1.04	1.05	1.1	1.06
Fifth	0.99	0.96	1.02	0.99
Averages	1.01	0.97	1.03	1.0

SEM = 0.1350; *p* = 0.6376 (type of supplement); *p* = 0.7411 (grazing cycle); *p* = 0.9926 (interaction).

**Table 9:** Final weight (kg) and carcass yield (%) according to supplementation.

Item	Treatments			SEM
	Control	Coco	Palm Kernel Cake	
^1^ Final weight *	631.4 ± 27.6	601.2 ± 28.3	610.6 ± 13.1	38.61
Carcass yield **	51.2 ± 2.5	49.3 ± 1.1	47.2 ± 0.9	2.34

* *p* = 0.6118 (type of supplement); ** *p* = 0.1418 (type of supplement). ^1^ Live weight refers to the last weighing of the animals on the farm.

## Data Availability

The data presented in this study are available upon reasonable request from the corresponding author.
